# Behavioural effects of task-relevant neuromodulation by rTMS on giving-up

**DOI:** 10.1038/s41598-021-01645-0

**Published:** 2021-11-18

**Authors:** Eri Miyauchi, Masahiro Kawasaki

**Affiliations:** Department of Intelligent Interaction Technologies, Faculty of Engineering, Information and Systems, University of Tsukuba, 1-1-1, Tennodai, Tsukuba, Ibaraki 305-8573 Japan

**Keywords:** Cognitive neuroscience, Psychology

## Abstract

Recent studies suggest that online repetitive transcranial magnetic stimulation (rTMS) can induce local entrainment of ongoing endogenous oscillatory activity during a task. This effect may impact cognitive performance, depending on the function of the oscillation. In this study, we aimed to investigate the effects of stimulation frequency and target location that are relevant to the cognitive processes of giving-up. We first investigated the correlations between the EEG oscillations and cognitive giving-up processes during problem-solving tasks (Experiment 1). We then conducted online rTMS to examine the frequency-dependent stimulation effects of rTMS on the performance of problem-solving tasks and ongoing oscillations (Experiment 2). The results of Experiment 1 suggested that the frontal theta rhythm is associated with the giving-up processes and that the frontal alpha rhythm is associated with problem-solving behaviour. Accordingly, we hypothesised that rTMS at the theta frequency would induce ongoing theta activity and accelerate the giving-up behaviour, while rTMS at the alpha frequency would induce ongoing alpha activity and slow down the giving-up behaviour in Experiment 2. The results showed that theta-frequency rTMS application induced an increase in theta amplitudes and shortened the giving-up response. Alpha-frequency rTMS application induced an increase in alpha amplitudes, but did not change giving-up responses. Considering the close resemblance between giving-up behaviour and rumination in depression, neuromodulation of cognitive giving-up processes may lead to a new intervention to treat depression by rTMS. Furthermore, this study strengthens the hypothesis that modulating task-relevant oscillations by rTMS could induce behavioural changes related to cognitive performance.

## Introduction

Giving-up refers to behaviours such as quitting to solve a problem^1^ or disengaging from goals that are too difficult to attain^2^. It is an adaptive behaviour that is associated with both mental and physical health, e.g. tendencies toward failure in goal disengagement are associated with depressive states^3^. Furthermore, van Randenborgh et al.^2^ demonstrated that rumination, a well-known risk factor for the onset and maintenance of depressive states^4^, plays a role in hindering adaptive giving-up behaviours. Therefore, intervening on cognitive function and behaviour of giving-up could lead to the treatment of rumination and depression; however, little is currently known about this in the field of cognitive and clinical neuroscience.

In the giving-up process, an individual tries to solve a problem, sets a goal to find a solution, though ultimately decides to disengage from the goal and stops trying to solve the problem, after goal-related and conflict thinking. Therefore, in the cognitive domain, giving-up is a process of making a decision evaluating the efforts requested and the benefits of solving the problem, involving cognitive processes such as decision-making, problem-solving, and cognitive control.

A growing body of literature recognises that specific frequency bands are linked to specific brain functions^5^. This observation led to electroencephalography (EEG) research that investigates the relationships between the human oscillatory brain activity and cognitive processes. Although there has been no detailed investigations on the neural oscillations in the giving-up process, an existing body of research on the related cognitive processes suggests that the frontal theta rhythm is the key mechanism underlying the giving-up process. The frontal regions and their oscillatory activity have been reported to be crucial for cognitive control in adaptive decision-making^6,7^. Specifically, prefrontal theta oscillations have been associated with the implementation of cognitive control, action monitoring, and flexible behaviour^7–10^. Moreover, theta amplitude increase in the anterior cingulate cortex (ACC) is associated with conflict resolution during cognitive tasks^11^, and the ACC, which is detected by the fronto-central electrodes, is related to the decision-making process, according to a functional imaging study^12^. In addition, a key point to consider is the involvement of alpha oscillations. Since giving-up is a way of solving a problem, cognitive giving-up processes should be associated with problem-solving. For example, the generation of original ideas has been linked to an increase of frontal alpha amplitude during creative problem-solving^13^. Furthermore, previous research has suggested that frontal alpha activity is related to divergent and convergent thinking^14^. It can therefore be assumed that in the problem-solving process, frontal alpha activity is important for generating possible courses of action to solve a problem, while frontal theta activity is important for decision-making in conflict resolution.

Recently, there has been an increasing interest in non-invasive neuromodulation techniques, such as transcranial magnetic stimulation (TMS). TMS can induce alterations in the neural activity underlying cognitive operations, and may thus provide to be a tool for cognitive and behavioural enhancement^15^. Particularly, the performance on a cognitive task can be modified by applying TMS in a repetitive paradigm (rTMS) over a central node of the brain network that is hypothesised to support a targeted cognitive function^16^. Furthermore, recent studies suggest that online rTMS, applied while a participant is engaged in a task, can induce local entrainment of ongoing endogenous oscillatory activity that impacts cognitive performance, and the effect may depend on the function of the oscillation^16–18^. Therefore, it seems reasonable to presume that the identification of the task-specific neural activity (i.e. areas and frequencies) of cognitive function with EEG, and subsequent rTMS application on the identified areas/frequencies would be beneficial for effective modulation of cognitive and behavioural performance. However, little is known about the effects of task-specific frequencies, especially when using an online rTMS paradigm^16^.

In the present study, we aimed to investigate the cognitive and behavioural effects of the stimulation frequency and target locations that are relevant with the cognitive processes of giving-up, by using combined TMS and EEG (TMS-EEG). To achieve this, we first investigated the oscillatory correlates of the cognitive processes of giving-up by measuring changes in EEG oscillations during a problem-solving task (Experiment 1). According to previous studies, it was hypothesised that the frontal theta rhythm would be associated with the giving-up processes^7–10^ and that the frontal alpha rhythm would be associated with the problem-solving behaviour^13,14,19^. Then, we explored the frequency-dependent stimulation effects of rTMS on the performance in the problem-solving task and ongoing oscillatory activity by online rTMS with EEG recording (Experiment 2). According to the results of Experiment 1, we hypothesised that rTMS at theta frequency would induce endogenous theta activity and accelerate the giving-up behaviour, while rTMS at alpha frequency would induce endogenous alpha activity and slow down the giving-up behaviour.

## Materials and methods

### Experiment 1

#### Participants

Twenty-two healthy participants (nine women and 13 men, aged 18–26 years; mean age 21.5 ± 2.6 years) completed the experiment. All participants reported normal visual acuity, hearing, and motor abilities. They also stated no history of: (1) any substance or medication use that could affect consciousness, (2) neurological disorders including seizure, stroke, or epilepsy, (3) asthma, (4) heart diseases, (5) neuropsychiatric disorders, (6) traumatic brain injury or surgery, (7) wearing a cardiac pacemaker or metal parts in the head, and (8) allergic scalp reactions. All participants gave written informed consent before participation. Data obtained from four participants were excluded from the analysis because of significant background EEG artefacts, leaving 18 for the final sample (eight women, 10 men, aged 18–26 years; mean age 21.4 ± 2.6 years). This study was approved by the Research Ethics Committee of the University of Tsukuba (2014R67) and conducted in accordance with the Declaration of Helsinki.

#### Task procedure

The experiment procedure was designed to capture the moment when participants gave up on problem solving. In a sound-proofed, electric-shielded room, participants sat on a chair, placed their chin on a chinrest, and wore earphones throughout the sessions. During each trial, participants were instructed to solve Japanese riddles presented on a computer display and to press keyboard buttons (keypress response) to indicate when they gave up on solving the riddles (giving-up) or when they solved the riddle (problem-solving). Each session lasted for 5 min and participants were allowed to work on the puzzles at their own pace within a session. The interval between the keypress and presentation of next riddle was 1.5 s. The riddles were selected from a royalty-free question bank on the Internet, and the difficulty of the problems was randomised. All participants completed three sessions. The inter-session intervals were at least 1 min long. Up to 30 riddles were prepared and distributed over three sessions.

#### EEG recording

Brain activity was measured using 27 active scalp electrodes (Fp1, Fp2, F7, F3, Fz, F4, F8, FC5, FC1, FC2, FC6, T7, C3, Cz, C4, T8, CP5, CP1, CP2, CP6, P7, P3, Pz, P4, P8, O1, and O2 electrodes) embedded in an electro cap (actiCAP) and BrainAmp DC equipment (Brain Products, Gilching, Germany). The sampling frequency was 1000 Hz. We used an online band-pass filter which was ranged from 0.1 to 50 Hz while EEG recordings. Reference electrodes were placed on the right and left mastoids. Vertical and horizontal electrooculography (EOG) was conducted by placing electrodes above and below the participant’s left eye, and electrodes at 1 cm from the right and left eyes. Electrode impedance was kept below 5 kΩ.

#### EEG analyses

We analysed EEG data with MATLAB software (R2015b, Mathworks Inc., Natick, MA, USA) to perform all the analyses. First, the EEG data was segmented into 8 s epochs (− 7.5 s to 0.5 s from the onset of keypress) in all sessions because approximately 7 s was the longest common duration before the key response for all data. Next, to reduce the artefacts which related to eye movements, we applied the infomax independent component analysis (ICA) to the EEG epochs, with the use of EEGLAB (Delorme and Makeig, 2004; Institute for Neural Computation, University of California, San Diego, CA, USA). We removed the ICA component which most correlated with the EOGs and recalculated the ICA-corrected EEG data by using regression with the remained ICA components. Finally, to get the time–frequency amplitudes, we conducted the wavelet analyses for the original EEG signals s (t) using Morlet’s function (t, f) in the time domain (SD σt) and the frequency domain (SD σf) around its centre frequency (f):

$$w(t,f) = (\sigma_{t} \sqrt \pi )^{{ - \frac{1}{2}}} \exp ( - t^{2} /2\sigma_{t}^{2} )\exp (i2\pi ft)$$

with $$\sigma_{f} = 1/(2\pi \sigma_{t} )$$.

We used a wavelet that was characterised by a constant ratio (f / σf = 7), with f ranging from 2–15 Hz (0.5-Hz steps). The amplitude was calculated by subtracting the baseline data measure in the intervals from the current keypress to the next riddle presentation (1.5-s time window) for each frequency band. To compare “giving-up” and “problem-solving” conditions, the Mann–Whitney U test was used.

### Experiment 2

#### Participants

Twenty-two healthy participants consented and participated in Experiment 2, and 21 participants completed the experiment. All participants reported normal visual acuity, hearing, and motor abilities. They also stated no history of: (1) any substance or medication use that could affect consciousness, (2) neurological disorders including seizure, stroke, or epilepsy, (3) asthma, (4) heart diseases, (5) neuropsychiatric disorders, (6) traumatic brain injury or surgery, (7) wearing a cardiac pacemaker or metal parts in the head, and (8) allergic scalp reactions. All participants gave written informed consent before participation. One participant reported a transient headache and withdrew from the experiment. We excluded one participant from the analysis because of too many EEG artefacts. Twenty participants were included in the analysis (eight women and 12 men, aged 18–39 years; mean age 23.5 ± 4.4 years). This study was approved by the Research Ethics Committee of the University of Tsukuba (2014R67-1, 2021R473) and performed in accordance with the Declaration of Helsinki.

#### Task procedure

**Figure 1:**
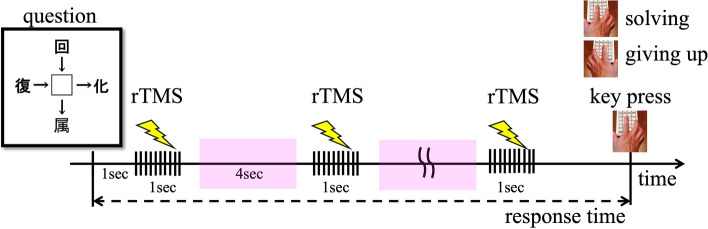
Experimental design. Seven seconds from the onset of keypress. The pink rectangles indicate the time window when participant’s averaged EEG amplitudes were calculated.

In a sound-proofed, electric-shielded room, participants sat on a chair, placed their chin on a chinrest, and wore earplugs throughout the sessions. During each trial, participants were instructed to solve Japanese crossword puzzles presented on a computer display and to press keyboard buttons when they gave up on solving the puzzles (giving-up) or when they solved the puzzles (problem-solving) (Fig. 1). Each session lasted for 5 min and participants were allowed to work on the puzzles at their own pace within a session. The problems were selected from a royalty-free question bank on the Internet. The order and the difficulty of the problems were randomised. All participants completed four sessions. Up to 30 puzzles were prepared and distributed over four sessions.

#### Application of rTMS delivery

rTMS was applied during the sessions of problem-solving tasks with the following conditions: alpha-TMS and theta-TMS for rTMS stimulation, and no-TMS and sham-TMS for the control; the order of conditions was counterbalanced across participants. We first conducted the task without rTMS (no-TMS) and identified the individual alpha and theta frequencies for each participant to determine the stimulation frequency of alpha and theta conditions, which were the peak amplitudes of 3–6 Hz and 9–13 Hz, respectively. For the theta- and alpha-TMS conditions, we applied TMS for 1 s at intervals of the 1 s divided by the determined theta and alpha stimulation frequencies, respectively (e.g. 250 ms in the case of 4 Hz). For the control, sham stimulation was conducted by applying TMS pulses at 15 cm from the top of the participant’s head (sham-TMS).

rTMS was applied using a figure-of-eight coil with a 70-mm wing diameter that was connected to a biphasic stimulator (Magstim Rapid MRS1000/50, Magstim Company Ltd., Whitland, United Kingdom). We used the flexible arm of a camera stand to fix the coil in the same position and direction for the duration of each session. We targeted the stimulation site as the frontal areas and located the TMS coil over the Fz electrode, according to the results of Experiment 1. The intensity of TMS was 95% of the motor threshold of each participant, which was defined as minimum TMS intensity to make his/her index finger twitch, before the task instruction was given^20,21^. We placed the TMS coil tangential to the scalp, with the handle pointing posterolaterally over the frontal area.

#### EEG recording

We used the same methods as the recordings for Experiment 1.

#### EEG analyses

We analysed only the Fz data. We used the same methods as the analyses for Experiment 1, expect for the rejection of the TMS artefacts and the epoch segmentations. The EEG data were segmented into 4-s time windows (from 0.5-s to 3-s after the last rTMS). To reduce the TMS-related artefacts, we removed the EEG data from −1 to 4 ms from TMS onset using linear interpolation, according to previous study^22^. The statistical differences were evaluated by the Mann–Whitney U tests. The statistics were corrected with the multiple comparison using the false discovery rate.

## Results

### Results of Experiment 1

#### Behavioural results

The response time (RT), which corresponds to the duration from stimulus onset to keypress response in the giving-up condition, was measured as the cognitive and behavioural response, and that in the problem-solving condition was measured as the control response. Due to the experimental design that the order and the difficulty of the presented problems were randomised and the approach to the problems was not controlled, giving-up and problem-solving conditions are not comparable and therefore the frequency of conditions was not measured. Participant-averaged RTs (± standard deviation [s.d.]) for giving-up and problem-solving conditions were as follows: 57.65 ± 13.60 s and 24.70 ± 8.38 s, respectively. There was a significant difference between conditions (z = − 3.306, p < 0.001).

#### EEG results

**Figure 2:**
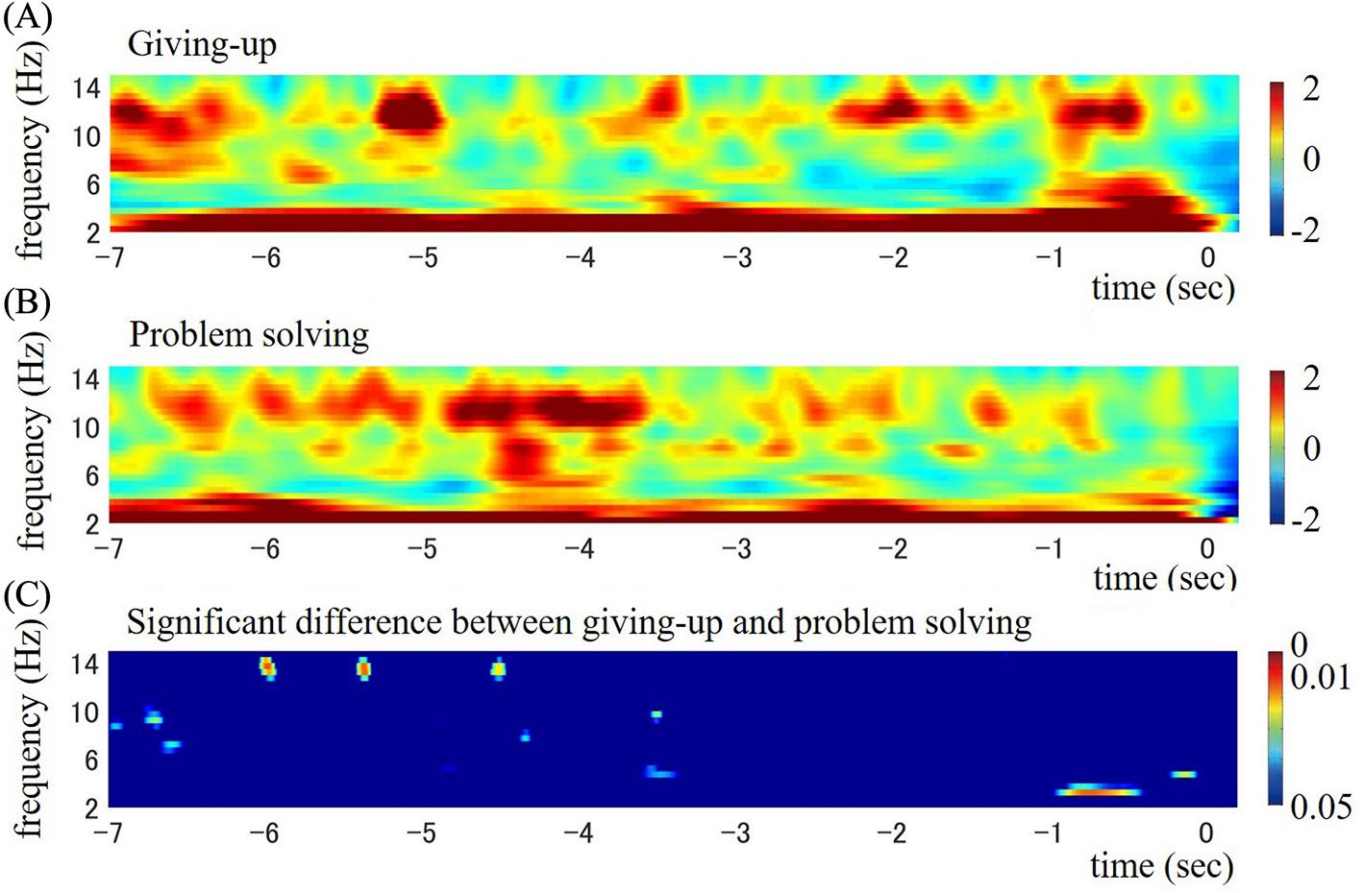
Time–frequency maps of frontal (Fz) amplitudes (μV) for seven seconds from the onset of keypress: (**A**) giving-up and (**B**) problem-solving conditions. (**C**) Statistically significant differences (p-values between 0.05 and 0.00) between the conditions.

To examine temporal activity of alpha and theta frequencies during the giving-up process, time–frequency analyses were conducted based on EEG data for the 7.0 s from the onset of stimulation to the keypress for each condition, and the data were compared to highlight the distinctive giving-up oscillatory behaviour. Figure 2 presents the participant-averaged time–frequency amplitudes of the Fz electrodes for giving-up (A), problem-solving (B), and their statistically significant differences between a p-value of 0.05 and 0.00 (C). There were intermittent increases in the alpha amplitude for both conditions, and an acute increase of the theta amplitude along with elevated alpha activity around 1 s before the keypress response was distinctive of giving-up.

### Results of Experiment 2

#### Modulatory effects on behaviour

**Figure 3:**
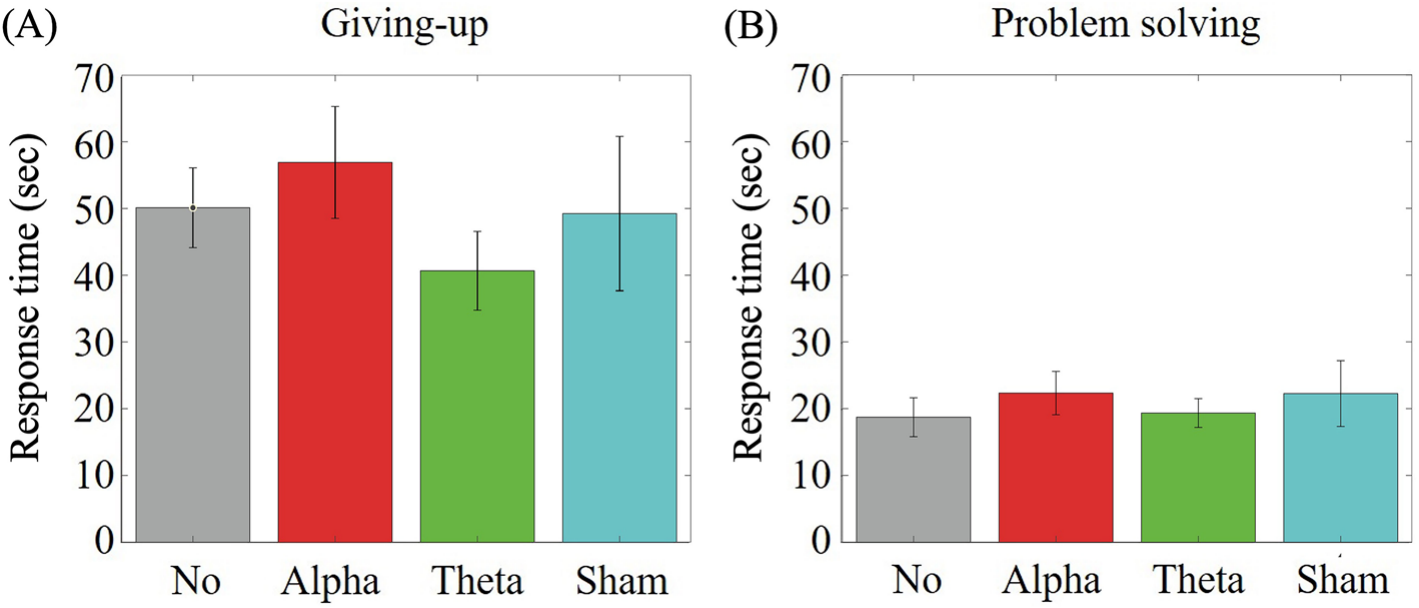
Modulatory effects of repetitive transcranial magnetic stimulation (rTMS) on (**A**) giving-up and (**B**) problem-solving behaviours in the rTMS condition.

The RT under no-TMS condition was considered as baseline response and sham-TMS condition was considered as control stimulation. Participant-averaged RTs (± s.d.) for giving-up under no-TMS, alpha-TMS, theta-TMS, and sham-TMS conditions were shown in Fig. 3 (A). There was no significant difference between sham-TMS and no-TMS condition (z = − 0.717, p = 0.474), suggesting that both conditions could be considered as control. There was a significant shortening RT in the theta-TMS condition compared to the no-TMS condition (z = − 2.165, p < 0.031), and RT was prolonged in the alpha-TMS condition compared to the no-TMS condition but not significantly (z = 0.971, p = 0.332).

Participant-averaged RTs (± s.d.) for problem-solving under no-TMS, alpha-TMS, theta-TMS, and sham-TMS conditions were shown in Fig. 3 (B). There was no significant modulatory effect on RT for problem-solving under alpha- and theta-TMS conditions compared to the no-TMS condition (z = 0.472, p = 0.720; z = 0.580, p = 0.562, respectively).

Due to the experimental design that the order and the difficulty of the presented problems were randomised and the approach to the problems was not controlled, giving-up and problem-solving conditions were not comparable and therefore the frequency of conditions was not measured.

#### Modulatory effects on oscillatory activity

**Figure 4:**
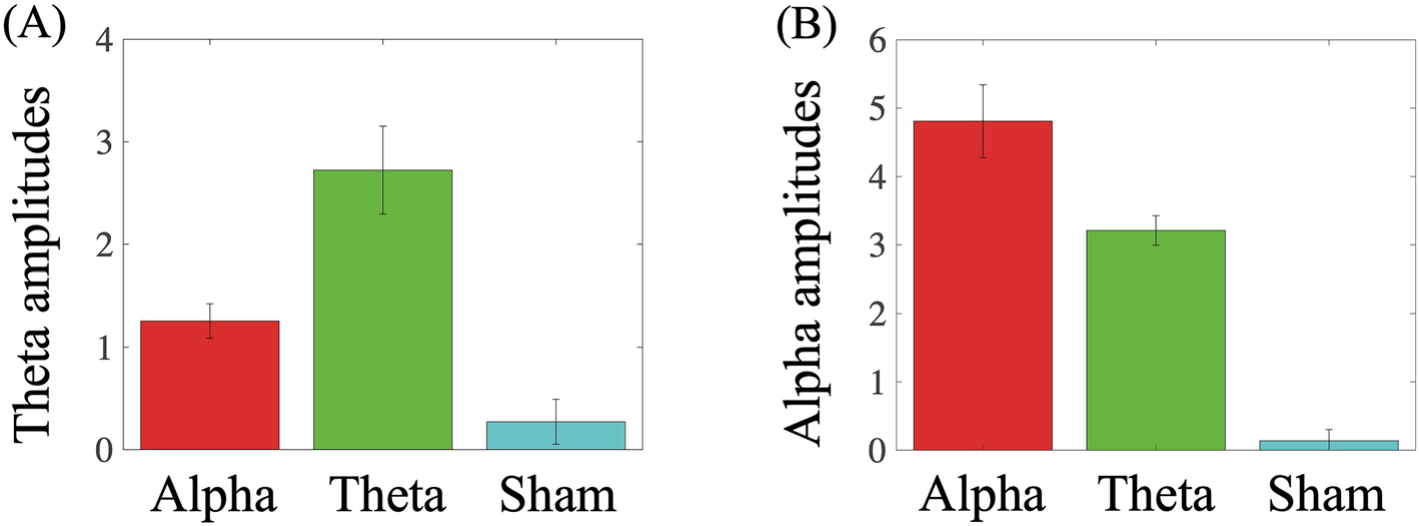
Modulatory effects of repetitive transcranial magnetic stimulation (rTMS) on (**A**) theta and (**B**) alpha amplitudes for the rTMS conditions.

In order to measure the modulatory effects of rTMS on oscillatory activity for giving-up, we calculated participant-averaged alpha and theta amplitudes in between stimulations (500 ms post-stimulation and 1 s pre-stimulation, as shown in Fig. 1) as rTMS-induced oscillatory amplitudes for alpha-, theta-, and sham-TMS conditions (Fig. 4). The greatest increase of the theta amplitude was induced by theta-TMS, and the greatest increase of the alpha amplitude was induced by alpha-TMS. The participant-averaged theta amplitude was significantly higher for the theta- and alpha-TMS conditions compared to the sham-TMS condition (z = 4.233, p < 0.001; z = 2.949, p < 0.004, respectively). Moreover, there was a significant difference between the theta- and alpha-TMS conditions (z = 2.744, p < 0.007). These results suggested that not only theta-TMS but also alpha-TMS has increasing effect of theta amplitude. The participant-averaged alpha amplitude was significantly different for the alpha-TMS condition compared to the sham- and theta-TMS conditions (z = 5.255, p < 0.001; z = 2.219, p < 0.027, respectively). Moreover, the alpha amplitude for the theta-TMS was significantly higher than those for the sham-TMS (z = 5.226, p < 0.001).

**Figure 5:**
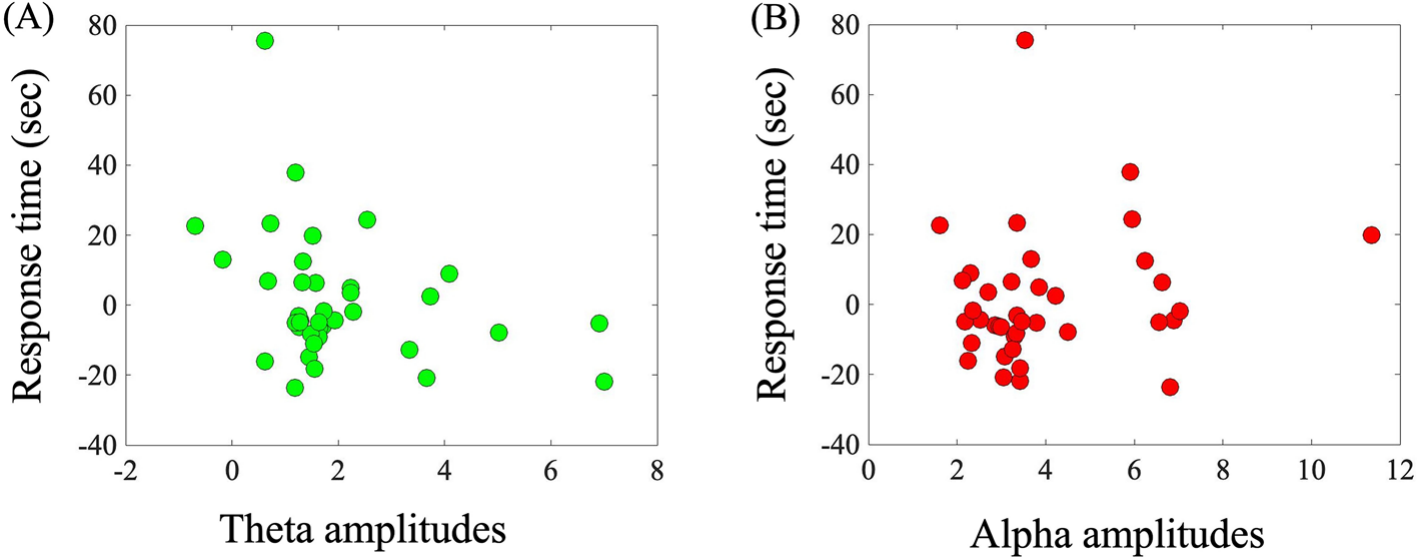
Relationship between response time (RT) and oscillatory amplitude induced by repetitive transcranial magnetic stimulation (rTMS) conditions. (**A**) Scatter plots between RT and the theta amplitude under the theta-TMS condition. (**B**) Scatter plots between RT and the alpha amplitude under the alpha-TMS condition.

In order to further illustrate the effects of stimulation frequency on rTMS-induced behaviour and oscillatory activity, we examined the relationship between rTMS-induced giving-up RTs and amplitudes for alpha- and theta-TMS conditions (Fig. 5). Our study showed that there was a significant correlation between RT and the theta amplitude (r = − 0.330, p < 0.044), suggesting that the greater increasing effect in the theta amplitude, the greater shortening effect in RT occurs (Fig. 5A). In contrast, there was no significant correlation between RT and the alpha amplitude (r = 0.170, p = 0.306; in Fig. 5B).

## Discussion

**Figure 6:**
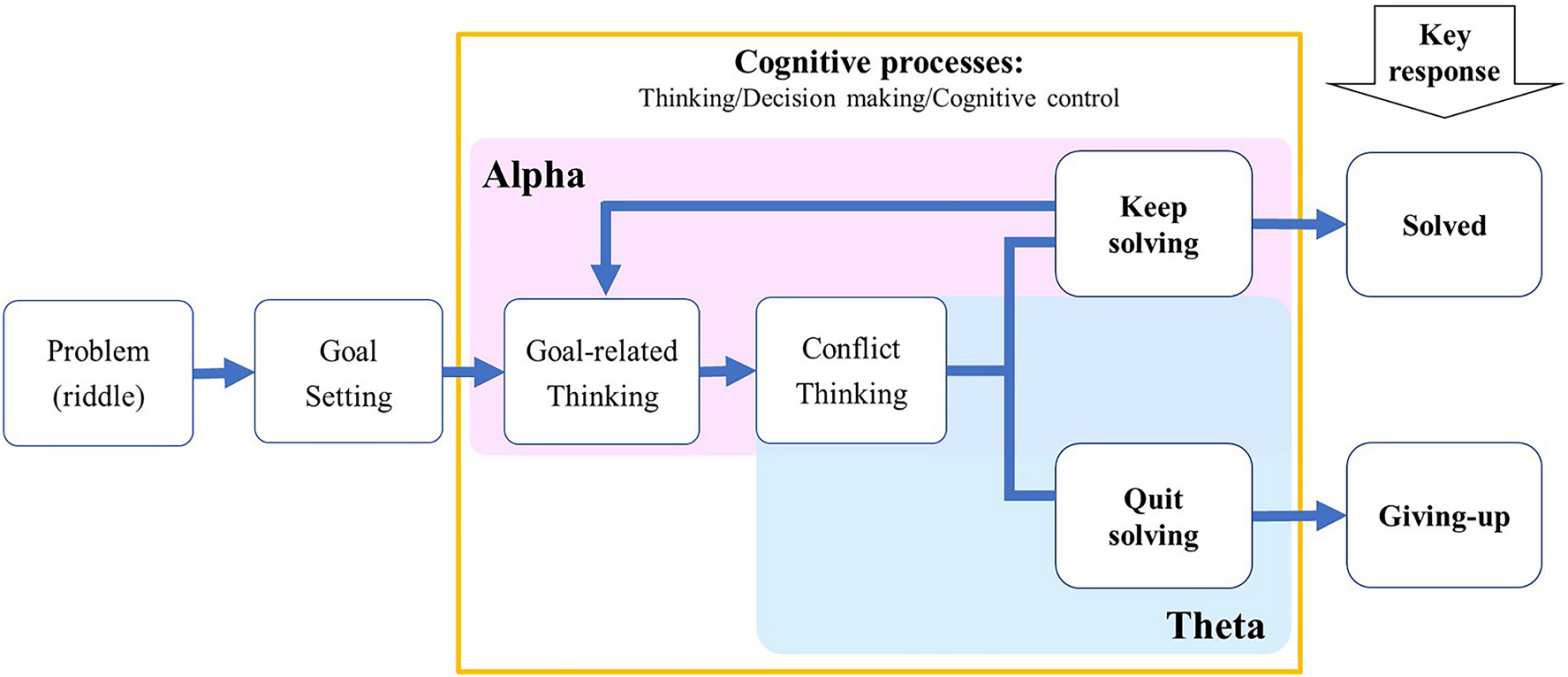
Hypothesised model for cognitive processes of problem-solving and giving-up.

This study was designed to determine the effects of the stimulation frequency and target location that are relevant to the cognitive processes of giving-up. We first investigated the correlations between the EEG oscillatory frequencies and the cognitive giving-up processes. Then we performed online rTMS to examine the task-relevant effects of rTMS on the performance in problem-solving tasks and ongoing oscillatory activity. It was hypothesised that the frontal theta rhythm would be associated with the giving-up process, while the frontal alpha rhythm would be associated with the problem-solving process (first hypothesis; Fig. 6). According to the results of Experiment 1, we hypothesised that rTMS at the theta frequency would induce ongoing theta activity and accelerate the giving-up behaviour, while rTMS at the alpha frequency would induce ongoing alpha activity and slow down the giving-up behaviour (second hypothesis).

We observed an acute increase in the theta amplitude immediately preceding the giving-up response, which was distinctive of giving-up and consistent with our first hypothesis. This finding may reflect the decision-making process to disengage from the goal and quit problem-solving as a type of conflict resolution, since the frontal theta amplitude is associated with the cognitive processes of decision-making, conflict resolution, and action monitoring^7,11,14^. The experiments also showed an intermittent increase in the alpha amplitude for both the giving-up and problem-solving conditions. This finding suggests the involvement of alpha oscillations in the cognitive processes of problem-solving, as hypothesised. These results are consistent with previous studies, which found that frontal alpha-band activity was related to creative problem-solving^13,14^. Moreover, the tonic increase of alpha activity has been reported to facilitate goal-directed behaviour^19^; therefore, the intermittent increase of alpha amplitude for both conditions possibly reflects engagement in the problem-solving tasks.

There was a significant shortening of giving-up RTs when rTMS induced an increase in theta activity. These results were consistent with our second hypothesis, confirming an association between the cognitive processes of giving-up and the theta amplitude in the frontal area, and that the increase in the theta activity is responsible for the decision to give up. In accordance with the present results, a recent TMS-EEG study has demonstrated that theta-burst stimulation to the frontal area altered the performance in decision-making tasks, compared to sham stimulation^23^. Contrary to expectations, this study did not find a significant prolongation of giving-up RTs despite that rTMS did induce an increase in alpha activity. This result may be explained by the fact that alpha oscillations are involved in various cognitive processes, as our results from Experiment 1 showed the increase of alpha activity in both the giving-up and problem-solving conditions, hence inducing specific cognitive behavioral change might be challenging. In addition, many studies have suggested that event-related synchronisation of alpha activity reflects upon cortical inhibition^24^, suggesting that there might be the involvement of different cognitive functions. To the best of our knowledge, this is the first study to investigate the neural oscillatory correlations of giving-up processes. Furthermore, the results demonstrated the effectiveness of using specific task-relevant stimulation frequency and target location for the modulation of cognitive and behavioural performance. It has been suggested that rumination, or repetitive negative thinking, is a risk factor and an important therapeutic target for depression^25,26^. Additionally, effective giving-up behaviour is associated with a lower tendency to ruminate^2^. Therefore, the study’s implications regarding effective means to encourage giving-up behaviour could be used as therapeutic options for depression, developing effective therapeutic use of rTMS. In fact, it has been demonstrated that rumination and emotional dysregulation with cognitive control in major depression is associated with the frontal regions and ACC^27,28^, suggesting the potential effectiveness of therapeutic rTMS approach targeting giving-up behaviour.

Several limitations in our study prevent the validation of the neuromodulatory effects of rTMS. Firstly, the small sample size and number of trials precluded us from conducting elaborate analyses or reach reliable conclusions regarding the results. In particular, we used the Mann–Whitney U tests for our statistical evaluation due to the limited number of trials for each rTMS condition. Therefore, we could not provide conclusive results regarding the effects of frequency-dependent stimulation. Further studies with larger sample sizes and trials could provide more definitive evidence regarding the frequency-specific effects of rTMS. Notably, recent studies have indicated the importance of calculating sample size and effect size in quantitative studies^29^.

Secondly, the present study did not employ a control site or frequency for comparing the rTMS paradigm. In addition, we only targeted and analysed the Fz electrode, which is considered to reflect neural activity of the ACC^12^. Although the target electrode was determined according to the previous research that the frontal area and the ACC is involved with adaptive decision-making [e.g.,^6,19^], additional targets which cover the pre-frontal regions [e.g.,^7,8^] could provide a deeper understanding of the effects of frequency-dependent stimulation. Therefore, there may be an alternative explanation for our results, suggesting the importance of detecting the task-relevant stimulation target site and frequency. Also, we did not use a neuronavigation system to locate the target site. Recent research that compared the behavioural effects of major targeting methods demonstrated that individualised functionally guided rTMS showed the most considerable effects, while scalp measurement localisation led to the smallest effects^30^. Considering that detecting a task-relevant stimulation target site is a method to individualise target location, using a functional neuronavigation could have increased the neuromodulatory effects found in this study. Therefore, future work should investigate effective targeting methods, such as the co-registration of functional magnetic resonance imaging with EEG for assessment and stimulation.

Thirdly, the present study did not evaluate whether blinding was successful under sham-condition. Although the blinding success of sham-stimulation is technologically challenging, as it depends on mimicking the acoustic and somato-sensory effects of active stimulation while ensuring that no brain stimulation occurs^31^, its importance has received more attention.

Lastly, handedness and smoking habit were not controlled in the study. The handedness effects on cortical excitability modulation in TMS studies are contradictory^32,33^; however, we cannot rule out the possibility of handedness effects on the results. Similarly, chronic nicotine consumption has been suggested to affect cortical excitability^34^. In future investigations, adding these for exclusion criteria could provide more definitive evidence.

## References

[1] Payne SJ, Duggan GB (2011). Giving up problem solving. Mem. Cognit..

[2] van Randenborgh A, Hüffmeier J, LeMoult J, Joormann J (2010). Letting go of unmet goals: Does self-focused rumination impair goal disengagement?. Motiv. Emo..

[3] Wrosch C, Miller GE, Scheier MF, de Pontet SB (2007). Giving up on unattainable goals: Benefits for health?. Personal. Soc. Psychol. Bull..

[4] Nolen-Hoeksema S, Wisco BE, Lyubomirsky S (2008). Rethinking rumination. Perspect. Psychol. Sci..

[5] Miniussi C, Thut G (2010). Combining TMS and EEG offers new prospects in cognitive neuroscience. Brain Topogr..

[6] Steinbeis N, Crone EA (2016). The link between cognitive control and decision-making across child and adolescent development. Curr. Opin. Behav. Sci..

[7] Wokke ME, Cleeremans A, Ridderinkhof KR (2017). Sure i’m sure: Prefrontal oscillations support metacognitive monitoring of decision making. J. Neurosci..

[8] Kawasaki M, Kitajo K, Yamaguchi Y (2010). Dynamic links between theta executive functions and alpha storage buffers in auditory and visual working memory. Eur. J. Neurosci..

[9] Cavanagh JF, Frank MJ (2014). Frontal theta as a mechanism for cognitive control. Trends Cogn. Sci..

[10] Cohen MX, Elger CE, Fell J (2008). Oscillatory activity and phase-amplitude coupling in the human medial frontal cortex during decision making. J. Cogn. Neurosci..

[11] Nigbur R, Cohen MX, Ridderinkhof KR, Stürmer B (2011). Theta dynamics reveal domain-specific control over stimulus and response conflict. J. Cogn. Neurosci..

[12] Botvinick MM, Cohen JD, Carter CS (2004). Conflict monitoring and anterior cingulate cortex: An update. Trends Cogn. Sci..

[13] Fink A (2009). The creative brain: Investigation of brain activity during creative problem solving by means of EEG and fMRI. Hum. Brain Mapp..

[14] Benedek M, Bergner S, Könen T, Fink A, Neubauer AC (2011). EEG alpha synchronization is related to top-down processing in convergent and divergent thinking. Neuropsychologia.

[15] Demirtas-Tatlidede A, Vahabzadeh-Hagh AM, Pascual-Leone A (2013). Can noninvasive brain stimulation enhance cognition in neuropsychiatric disorders?. Neuropharmacology.

[16] Beynel L (2019). Effects of online repetitive transcranial magnetic stimulation (rTMS) on cognitive processing: A meta-analysis and recommendations for future studies. Neurosci. Biobehav. Rev..

[17] Thut G, Miniussi C (2009). New insights into rhythmic brain activity from TMS–EEG studies. Trends Cogn. Sci..

[18] Thut G (2011). Rhythmic TMS causes local entrainment of natural oscillatory signatures. Curr. Biol..

[19] Dockree PM, Kelly SP, Foxe JJ, Reilly RB, Robertson IH (2007). Optimal sustained attention is linked to the spectral content of background EEG activity: Greater ongoing tonic alpha (∼10 Hz) power supports successful phasic goal activation. Eur. J. Neurosci..

[20] Rossi S (2009). Safety, ethical considerations, and application guidelines for the use of transcranial magnetic stimulation in clinical practice and research. Clin. Neurophysiol..

[21] Zarkowski P, Navarro R, Pavlicova M, George MS, Avery D (2009). The effect of daily prefrontal repetitive transcranial magnetic stimulation over several weeks on resting motor threshold. Brain Stimul..

[22] Veniero D, Bortoletto M, Miniussi C (2009). TMS-EEG co-registration: On TMS-induced artifact. Clin. Neurophysiol..

[23] Si Y (2019). Different decision-making responses occupy different brain networks for information processing: A study based on EEG and TMS. Cereb. Cortex.

[24] Klimesch W (2012). Alpha-band oscillations, attention, and controlled access to stored information. Trends Cogn. Sci..

[25] McLaughlin KA, Nolen-Hoeksema S (2011). Rumination as a transdiagnostic factor in depression and anxiety. Behav. Res. Ther..

[26] McEvoy PM, Watson H, Watkins ER, Nathan P (2013). The relationship between worry, rumination, and comorbidity: Evidence for repetitive negative thinking as a transdiagnostic construct. J. Affect. Disord..

[27] Lantrip C, Gunning FM, Flashman L, Roth RM, Holtzheimer PE (2017). Effects of transcranial magnetic stimulation on the cognitive control of emotion: Potential antidepressant mechanisms. J. ECT.

[28] Nejad AB, Fossati P, Lemogne C (2013). Self-referential processing, rumination, and cortical midline structures in major depression. Front. Hum. Neurosci..

[29] Sullivan GM, Feinn R (2012). Using Effect Size—or Why the P Value Is Not Enough. J. Grad. Med. Educ..

[30] Sack AT (2009). Optimizing functional accuracy of TMS in cognitive studies: A comparison of methods. J. Cogn. Neurosci..

[31] Duecker F, Sack AT (2015). Rethinking the role of sham TMS. Front. Psychol..

[32] Cahn SD, Herzog AG, Pascual-Leone A (2003). Paired-pulse transcranial magnetic stimulation: Effects of hemispheric laterality, gender, and handedness in normal controls. J. Clin. Neurophysiol..

[33] Reid CS, Serrien DJ (2012). Handedness and the excitability of cortical inhibitory circuits. Behav. Brain Res..

[34] Grundey J (2013). Cortical excitability in smoking and not smoking individuals with and without nicotine. Psychopharmacology.

